# Substitution of lead with tin suppresses ionic transport in halide perovskite optoelectronics†

**DOI:** 10.1039/d3ee03772j

**Published:** 2023-11-27

**Authors:** Krishanu Dey, Dibyajyoti Ghosh, Matthew Pilot, Samuel R. Pering, Bart Roose, Priyanka Deswal, Satyaprasad P. Senanayak, Petra J. Cameron, M. Saiful Islam, Samuel D. Stranks

**Affiliations:** a Cavendish Laboratory, University of Cambridge Cambridge UK sds65@cam.ac.uk; b Department of Materials Science and Engineering and Department of Chemistry, Indian Institute of Technology Delhi Hauz Khas India; c Department of Chemistry, University of Bath Bath UK chppjc@bath.ac.uk; d Department of Materials, Loughborough University Loughborough UK; e Department of Chemical Engineering and Biotechnology, University of Cambridge Cambridge UK; f Department of Physics, Indian Institute of Technology Delhi Hauz Khas India; g Nanoelectronics and Device Physics Lab,School of Physical Sciences, National Institute of Science Education and Research, HBNI, Jatni India; h Department of Materials, University of Oxford Oxford UK saiful.islam@materials.ox.ac.uk

## Abstract

Despite the rapid rise in the performance of a variety of perovskite optoelectronic devices with vertical charge transport, the effects of ion migration remain a common and longstanding Achilles’ heel limiting the long-term operational stability of lead halide perovskite devices. However, there is still limited understanding of the impact of tin (Sn) substitution on the ion dynamics of lead (Pb) halide perovskites. Here, we employ scan-rate-dependent current–voltage measurements on Pb and mixed Pb–Sn perovskite solar cells to show that short circuit current losses at lower scan rates, which can be traced to the presence of mobile ions, are present in both kinds of perovskites. To understand the kinetics of ion migration, we carry out scan-rate-dependent hysteresis analyses and temperature-dependent impedance spectroscopy measurements, which demonstrate suppressed ion migration in Pb–Sn devices compared to their Pb-only analogues. By linking these experimental observations to first-principles calculations on mixed Pb–Sn perovskites, we reveal the key role played by Sn vacancies in increasing the iodide ion migration barrier due to local structural distortions. These results highlight the beneficial effect of Sn substitution in mitigating undesirable ion migration in halide perovskites, with potential implications for future device development.

## Abstract 2

Mobile ions cause deterioration in both device performance and stability of lead halide perovskite devices. This study provides direct evidence for substantial suppression of ionic migration effects in tin-containing perovskite compositions.

## Graphical abstract

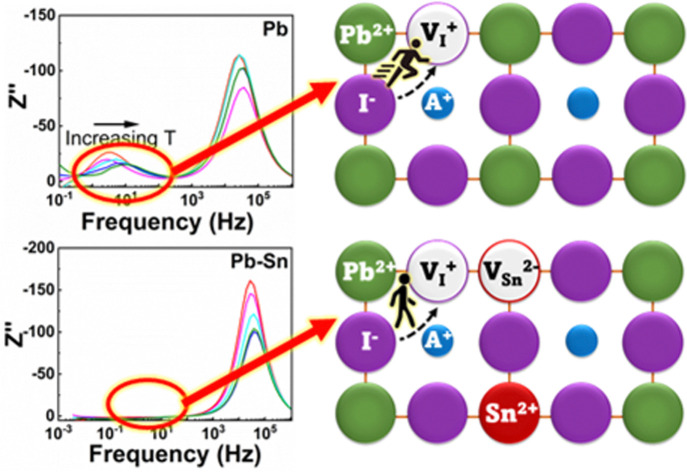

**Broader context**

Organic–inorganic halide perovskites are at a critical juncture in their journey towards commercialization, where limited operational stability of devices to external stressors (such as light and bias) remains the single biggest challenge that needs to be addressed. One of the key drivers of such instability is ionic migration, which is also believed to be responsible for the widely observed hysteresis in current–voltage characteristics of perovskite solar cells and partially for the efficiency roll-off at high injection currents in perovskite LEDs. While extensive studies on understanding and mitigating ion migration effects have been conducted on lead perovskite devices, similar efforts on their tin-containing counterparts are scarce. In this work, using a combination of experimental measurements on operating solar cells, we provide direct evidence for the suppressed ionic transport in mixed Pb–Sn perovskites compared to their Pb-only analogues. Furthermore, by conducting atomistic *ab initio* simulations, we attribute such observations to the presence of tin vacancies in tin-containing perovskites, which are found to increase the iodide ion migration barrier due to local structural distortion. Our work highlights the often-overlooked brighter aspects of tin in halide perovskites, which can be further leveraged for extending the operational stability of a variety of perovskite-based energy devices, including tandem solar cells, LEDs, photo- and X-ray detectors, among others.

## Introduction

Lead halide perovskites have shown a remarkable run in photovoltaic applications, with single junction solar cell efficiencies reaching close to 26% and tandem efficiencies (with Si) eclipsing 33%.^1^ At the same time, external quantum efficiencies of perovskite LEDs have ascended to more than 20% for green, red and infrared emission.^2^ In addition to their favourable optoelectronic properties, including high absorption coefficient,^3^ large ambipolar carrier diffusion length^4^ and facile bandgap tunability,^5^ these developments in the device performance of halide perovskites have also been aided by the relative ease of processing of these materials using inexpensive solution-based methods that require low thermal budgets.^6^ However, unlike more conventional semiconductors like silicon or III–V materials (*e.g.* GaAs), halide perovskites are ‘soft’ semiconductors where the constituent ions (*i.e.* A^+^, B^2+^ and X^−^ ions in the standard ABX_3_ stoichiometry) migrate in response to external stimuli, such as electrical voltage, temperature and light.^7–11^ Such ionic transport is mediated by the presence of various point and extended defects that are inevitably formed during the low-temperature growth of polycrystalline films on non-epitaxial substrates,^12^ and represents one of the biggest challenges that needs to be tackled for demonstrating prolonged operational stability in solar cells and LEDs.

The manifestation of ion migration in lead (Pb) perovskite devices was first observed in the appearance of apparent large low-frequency dielectric constant and pronounced current–voltage hysteresis in perovskite solar cells.^13,14^ Since then, a general consensus points towards the halide ions as the dominant mobile ionic species under standard operational conditions.^8,15–17^ These mobile ions have been shown to not only result in open circuit voltage gain and short circuit current loss under light soaking in perovskite solar cells,^18,19^ but also affect the long-term performance of perovskite solar cells and LEDs.^20–23^ In recent years, various strategies have been demonstrated to mitigate such effects of ion migration. For example, metal doping in Pb halide perovskites has been attempted as a mitigation measure for ion migration in operational devices.^24–28^ Mixing differently sized organic cations (in the A-site of 3D perovskites and/or A′ site of 2D perovskites) has also been another effective approach to hinder the ion transport and extend device operational stability.^29–32^ In addition, modulating grain sizes as well as passivating defects along grain boundaries have all achieved promising results.^33–36^ Similarly, interface engineering in Pb-based perovskite solar cells has also led to efficient suppression of ionic migration effects.^37,38^ although the atomistic mechanisms are often not fully characterised.

Despite these efforts, there is still a limited understanding on the role of tin (Sn) substitution on the dynamics of ion migration in Pb halide perovskite optoelectronic devices. This is of importance given the recent fast pace developments in the fields of Sn and mixed Pb–Sn perovskite solar cells towards, among others, reduced lead and tandem solar cell applications.^39–41^ Although the recent report from Ighodalo *et al.* seems to suggest negligible (or complete absence of) ion migration in pure-Sn perovskites,^42^ their conclusions were based on lateral device structures and all-inorganic pure-bromide compositions, both of which are not directly relevant for hybrid perovskite-based optoelectronic applications with vertical charge transport (such as solar cells and LEDs). For example, such claims are found to be contradictory to the work from Thiesbrummel *et al.*,^19^ where the presence of ionic migration effects was clearly evident in organic–inorganic mixed Pb–Sn iodide perovskite solar cells. Thus, it is vital to fully rationalize and understand the impact of Sn substitution on the dynamics of ion migration in Pb halide perovskite optoelectronic devices.

In this work, using electrical measurements on solar cells, we show that Sn-containing Pb-based hybrid perovskites exhibit slower ionic diffusion when compared to their Pb-only analogues. With further insights obtained from first-principles calculations, we attribute these observations to the increased iodide migration barriers in Sn-containing perovskites due to the structural distortion associated with Sn vacancy defects. The key conclusions derived from this study are applicable to various Sn-containing perovskite optoelectronic devices and will help guide future materials and device development.

## Results & discussion

### Material properties and device performance

**Fig. 1:**
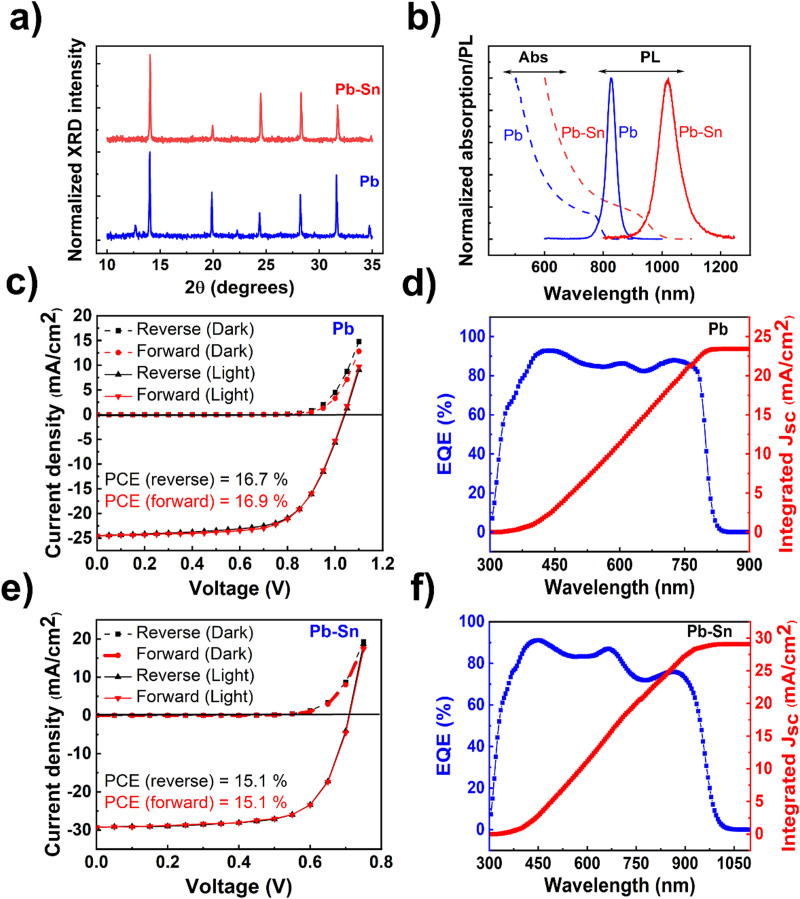
Material and device characterization. (a) XRD patterns of Pb and Pb–Sn perovskite thin films. (b) Absorption spectra (obtained from UV-visible-near-infrared spectroscopy) and photoluminescence spectra of Pb and Pb–Sn perovskite thin films. (c) Light (under AM 1.5G illumination) and dark *J*–*V* scans of a characteristic Pb perovskite solar cell (with 2PACz HTL) with a scan rate of 100 mV s^−1^. (d) External quantum efficiency (EQE) spectra of the corresponding Pb perovskite solar cell. (e) Light (under AM 1.5G illumination) and dark *J*–*V* scans of a characteristic Pb–Sn perovskite solar cell (with PEDOT:PSS HTL) with a scan rate of 100 mV s^−1^. (f) EQE spectra of the corresponding Pb–Sn perovskite solar cell. Here, ‘Pb’ refers to FA_0.85_Cs_0.15_PbI_3_ and ‘Pb–Sn’ refers to FA_0.85_Cs_0.15_Pb_0.5_Sn_0.5_I_3_. Optical bandgaps of the perovskite films were calculated from the absorption spectra using the Tauc-plot method. For PL measurements, a continuous-wave 405 nm laser was used as the excitation source. All the solar cell measurements were performed in air on encapsulated devices.

The perovskite films and devices used in this study are based on solution-processed methylammonium (MA)-free compositions, *viz.* FA_0.15_Cs_0.15_PbI_3_ (henceforth referred to as ‘Pb perovskite’) and FA_0.85_Cs_0.15_Pb_0.5_Sn_0.5_I_3_ (henceforth referred to as ‘Pb–Sn perovskite’), where FA refers to the formamidinium cation. X-ray diffraction (XRD) measurements performed on the films (Fig. 1a) reflect the standard perovskite crystalline peaks observed for Pb and Pb–Sn perovskites, in addition to a small PbI_2_ peak (at ∼12.7°) appearing for the Pb perovskite. Fig. 1b shows the absorption spectra of the corresponding films, demonstrating bandgaps (*E*_g_) of 1.53 eV and 1.24 eV respectively for Pb and Pb–Sn perovskites. Photoluminescence (PL) spectra are also plotted alongside in Fig. 1b, revealing peak emission at 827 nm (FWHM = 38 nm) and 1022 nm (FWHM = 80 nm) for the respective perovskites. Scanning electron microscopy (SEM) images of the films (Fig. S1, ESI†) yield grain sizes of 248 ± 102 nm and 308 ± 93 nm for Pb and Pb–Sn perovskites respectively.

We then fabricated perovskite solar cells with p–i–n configuration with the architecture: ITO/hole transporting layer (HTL)/perovskite/fullerene (C_60_)/bathocuproine (BCP)/Cu. Through careful optimization (Fig. S2–S5, ESI†), [2-(9*H*-Carbazol-9-yl)ethyl] phosphonic acid (2PACz) and PEDOT:PSS were found to be the optimum HTLs for our Pb and Pb–Sn perovskite devices. For Pb perovskite solar cells, *J*–*V* scans (reverse and forward) of a characteristic device (with 2PACz HTL) is displayed in Fig. 1c, with open circuit voltage (*V*_oc_) of 1.04 V, short circuit current density (*J*_sc_) of 24.5 mA cm^−2^, fill factor (FF) of 66.4% and power conversion efficiency (PCE) of 16.9%. The average photovoltaic parameters are summarized in Table 1 (see Fig. S2 for statistical distributions, ESI†). Moreover, the integrated *J*_sc_ obtained from external quantum efficiency measurements (Fig. 1d) is 23.4 mA cm^−2^, which agrees well with the average *J*_sc_ of 23.9 mA cm^−2^ obtained from *J*–*V* measurements. On the other hand, Fig. 1e shows *J*–*V* scans of a characteristic Pb–Sn perovskite solar cell (with PEDOT:PSS HTL), with the average of photovoltaic parameters given in Table 1 (device statistics in Fig. S5, ESI†). The corresponding EQE spectrum is shown in Fig. 1f, which gives an integrated *J*_sc_ of 29.1 mA cm^−2^ and agrees closely with that obtained from *J*–*V* measurements (28.8 mA cm^−2^). While much thicker (>800 nm) absorber layers are ideal for minimizing optical losses in Pb–Sn perovskite solar cells,^43^ we did not obtain any appreciable improvement in the efficiency by increasing the concentration of perovskite solution from 1.35 M to 1.8 M (device statistics in Fig. S6, ESI†). We have intentionally not used any defect passivating additives in the perovskite solution or as post-deposition surface treatments in the fabricated device stacks because of their synergistic influence on ion migration. Therefore, conclusions derived from this study are applicable in general for various Sn-containing perovskite optoelectronic devices.

**Table 1:** Average photovoltaic parameters of Pb and Pb–Sn perovskite solar cells

Device	*V*_oc_ (V)	*J*_sc_ (mA cm^−2^)	FF (%)	PCE (%)
Pb (2PACz)	1.02 ± 0.04	23.9 ± 1.0	64.5 ± 2.2	15.6 ± 1.3
Pb–Sn (PEDOT)	0.77 ± 0.04	28.9 ± 0.9	67.8 ± 4.7	14.3 ± 1.1

### Ionic transport properties from scan-rate dependent measurements

**Fig. 2:**
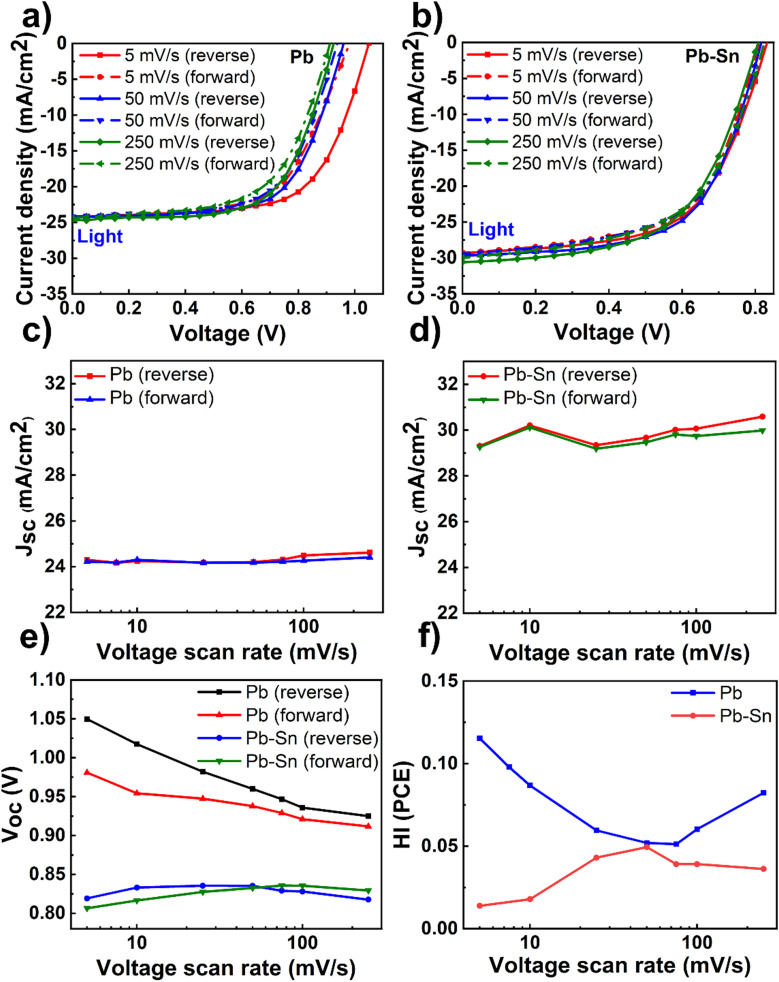
Scan-rate dependent *J*–*V* measurements. Light *J*–*V* scans of (a) Pb and (b) Pb–Sn perovskite solar cells at three scan rates: 5 mV s^−1^, 50 mV s^−1^ and 250 mV s^−1^. (c) *J*_sc_ of Pb perovskite solar cells as a function of scan rates in the reverse and forward scans. (d) *J*_sc_ of Pb–Sn perovskite solar cells as a function of scan rates in the reverse and forward scan. (e) Open circuit voltage of Pb and Pb–Sn perovskite solar cells as a function of scan rates in the reverse and forward scans. (f) Hysteresis index (HI) in PCE of Pb and Pb–Sn perovskite solar cells as a function of scan rates. Note that optimized 2PACz and PEDOT:PSS were used as HTLs respectively for Pb and Pb–Sn perovskite solar cells.

Having optimized material and device fabrication, we investigated the nature and extent of the role of ion transport in solar cells comprised of Pb and Pb–Sn perovskites. We performed *J*–*V* measurements over a range of scan rates from 5 mV s^−1^ to 250 mV s^−1^ to capture any time-dependent performance changes in the devices. In these measurements, the reverse scan is followed by the forward scan at a given scan rate and then the scan rate is changed to repeat this routine. We did not lower the scan rate below 5 mV s^−1^ due to long measurement times (tens of minutes) and thus potential degradation of the devices due to bias stress, especially in dark conditions. Light *J*–*V* scans of Pb and Pb–Sn perovskite solar cells at three representative scan rates of 5 mV s^−1^, 50 mV s^−1^ and 250 mV s^−1^ are shown in Fig. 2a and b (dark *J*–*V* scans in Fig. S7, ESI†), with the corresponding variation in the PCE of solar cells as a function of scan rates shown in Fig. S8 (ESI†).

We display the variation of *J*_sc_ as a function of scan rates in the Pb and Pb–Sn cells in Fig. 2c and d, which shows an overall increase in *J*_sc_ (during both reverse and forward scans) with increasing scan rates from 5 mV s^−1^ to 250 mV s^−1^ for both Pb and Pb–Sn perovskite solar cells. Such a trend can be rationalized by the fact that at slow scan rates, diffusing ions have enough time to react to the changes in voltage and hence these can move their equilibrium positions from the bulk (assuming roughly homogenous distribution at *V*_oc_) towards the interfaces with the charge transport layers. Such movement of ions causes screening of the internal built-in field at short circuit, leading to a lowering of *J*_sc_.^19^ These observations point to the presence of mobile ions in both kinds of solar cells. Furthermore, a consistent increase in *V*_oc_ is also observed by lowering the scan rates (during both reverse and forward scans) for the Pb device (Fig. 2e), which may also originate from the prolonged light soaking effects on ion migration as observed by Herterich *et al.*^44^ However, no such increase in *V*_oc_ is observed for Pb–Sn devices.

Next, we calculated the variation of hysteresis index (HI) as a function of scan rates (Fig. 2f) for both Pb and Pb–Sn perovskite solar cells, where HI in PCE is defined as 
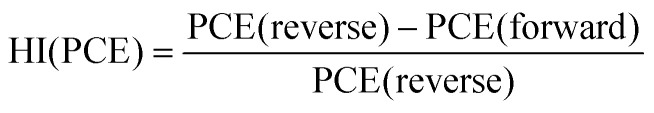
.^45^ In the regime of scan rates <50 mV s^−1^, we observe a significant uptick in the HI (PCE) of Pb devices, while a relatively flat response is seen for their Pb–Sn analogues. Such a phenomenon of increasing hysteresis at lower scan rates for Pb perovskites is in agreement with reported drift-diffusion modelling on p–i–n perovskite solar cells involving ionic migration effects.^46,47^

While interface recombination and band alignment with charge transporting layers can also influence hysteresis in operating solar cells, our choice of appropriate HTLs (2PACz for Pb perovskite devices and PEDOT:PSS for Pb–Sn perovskite devices) bring the two perovskite systems closest to their optimum performance with respect to *V*_oc_ (which is affected by the non-radiative recombination of carriers at the bulk and the interfaces) and FF (which is impacted by the band alignment and corresponding charge extraction at the interfaces). Thus, we argue that our scan-rate-dependent *J*–*V* hysteresis measurements are predominantly influenced by ionic migration effects, thereby indicating that the ionic transport in Pb–Sn devices is significantly slowed down as compared to that in Pb devices.

### Ionic transport properties from impedance spectroscopy

**Fig. 3:**
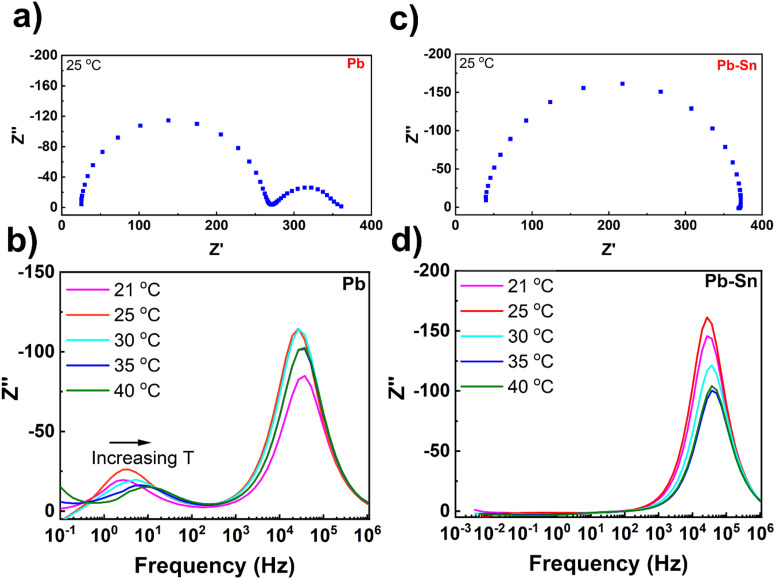
Impedance spectroscopy measurements. (a) Nyquist plot of a Pb perovskite solar cell measured under illumination with a 470 nm LED at open circuit, while the cell was held at 25 °C throughout the measurement. (b) Plots of the imaginary impedance against frequency for the corresponding Pb perovskite solar cell within a range of temperatures (21–40 °C). (c) Nyquist plot of a Pb–Sn perovskite solar cell measured under illumination at open circuit, while the cell was held at 25 °C throughout the measurement. (d) Plots of the imaginary impedance against frequency for the corresponding Pb–Sn perovskite solar cell within a range of temperatures (21–40 °C). All data were assessed through a Kramers–Kronig transformation which shows that the measured data are valid within these frequencies (Fig. S11, ESI†).

To further understand the extent and impact of ionic diffusion, we employ impedance spectroscopy, which is a powerful technique to disentangle the ionic and electronic response in perovskite device stacks due to the characteristically different time scales of the two phenomena.^48–50^ We carried out temperature-controlled impedance spectroscopy on Pb and Pb–Sn perovskite solar cells at open circuit under illumination with a blue 470 nm LED (Fig. 3). To ensure the cells were stable during the measurements, they were loaded in a gas-tight device holder in a N_2_ glovebox and also measured under nitrogen. Furthermore, to eliminate any complexity in the impedance response arising due to different HTLs, we used 2PACz as the common HTL for both Pb and Pb–Sn perovskite devices. Moreover, 2PACz-based devices were also found to be more stable under long-term measurements than the PEDOT-based devices.

Fig. 3a shows the impedance spectra of a representative Pb perovskite solar cell measured at 25 °C over a frequency range of 100 mHz to 1 MHz. The Nyquist plot shows two-semi-circles, with a high frequency (hf) and lower frequency (lf) process clearly visible. The high frequency response gives a characteristic lifetime of 6.2 μs, which corresponds to a geometric capacitance (*C*_geo_) of 26 nF and a recombination resistance (*R*_recomb_) of 240 Ω. The time constant for the lower frequency process at 25 °C was 45 ms, which is consistent with the time constant we have previously measured for the ‘lf’ response attributed to ion diffusion inside perovskite crystallites.^30,51^ Increasing the cell temperature from 21 °C to 40 °C results in an increase of characteristic frequencies for the ‘lf’ response (Fig. 3b), indicating temperature-activated ionic transport in the devices. By fitting the low frequency time constants as a function of temperatures (Fig. S9, ESI†), an activation energy of 0.52 eV can be obtained which is in the range typically measured for iodide ion migration.^30^

In contrast, the impedance spectra of the Pb–Sn cell at 25 °C (Fig. 3c) interestingly consists of only the ‘hf’ semicircle, with no evidence of the ‘lf’ ion mediated response. The ‘hf’ response has a characteristic lifetime of 5.8 μs, corresponding to a *R*_recomb_ of 340 Ω and a *C*_geo_ of 17 nF, which are similar to those obtained for the Pb cells. Moreover, even after increasing the cell temperature to 40 °C, it was still not possible to resolve any ‘lf’ response for the Pb–Sn device, even down to frequencies close to 1 mHz (Fig. 3d). Furthermore, a similar trend is found for the Pb–Sn perovskite cells when using PEDOT:PSS HTL instead of 2PACz (Fig. S10, ESI†). Therefore, these results indicate that ionic migration in these Pb–Sn cells is much slower than in the equivalent Pb perovskites. This suggests an activation energy for ion migration substantially higher than that obtained for our Pb perovskites (*i.e.* 0.52 eV) and we would likely have to go to higher temperatures or lower frequencies to see the response. This is not practical as typical perovskite cells are not stable for long periods at elevated temperatures or during the very long measurement times needed to measure at frequencies below 1 mHz. In fact, extreme care needs to be taken when interpreting the low frequency response of all perovskite cells due to additional features that can be introduced by degradation.

### Atomistic migration mechanisms through *ab initio* simulations

To complement the experimental work with atomic-scale insights into ion migration mechanisms, we also performed systematic density functional theory (DFT) calculations (see details of the computational methodology in Methods). Previous studies by us and other groups have demonstrated that halide ions are the dominant migrating species *via* a vacancy mechanism in lead-halide perovskites.^8,17,30,52^ Here, we investigate the migration pathways and energy barriers for iodide vacancy migration in Pb, mixed Pb–Sn and Sn perovskites.

To examine the impact of Sn substitution, we first focused on the FAPbI_3_ perovskite structure, which allowed us to probe trends in ion migration energies in a systematic manner. Due to the tetragonal distortion in the lattice, there are two inequivalent iodide sites in FAPbI_3_ and, consequently, we find that the two most probable pathways for iodide vacancy migration are equatorial–equatorial and axial–equatorial mechanisms, for which the calculated migration barriers are 0.34 eV and 0.45 eV, respectively (Fig. S12, ESI†). These results are in good agreement with previous experimental and computational work on Pb-based systems.^13,30^ We anticipate the axial–equatorial iodide migration as the rate-determining step for long-range diffusion in the material, and focus on this pathway for the rest of the study. In addition, following similar methodology, our simulations on FASnI_3_ find an activation energy of 0.36 eV for the axial–equatorial pathway of iodide migration.

We then explored iodide migration and associated energy barriers in the mixed-metal system FAPb_0.5_Sn_0.5_I_3_. Due to such B-metal alloying, there are two inequivalent pathways for axial–equatorial migration involving Pb-centred and Sn-centred iodide diffusion (see Fig. S13, ESI†), for which we find energy barriers of 0.43 eV and 0.47 eV, respectively. Thus, our simulations suggest that B-metal alloying alone does not have any major impact on iodide migration barriers in these Pb–Sn halide perovskites.

It is known that there is a significant population of Sn vacancy defects in Sn-containing perovskites due to their low formation energies.^53–55^ Moreover, thermodynamic ionization levels of these defects lie close to the valence band maximum for mixed Pb–Sn perovskites, while they lie inside the valence band for pure-Sn perovskites. Therefore, these Sn vacancies can be easily ionized, thereby resulting often in the unintentional hole doping in Sn-containing perovskites.^53^ If left uncontrolled, excessive hole doping can affect the short circuit current and fill factor of the fabricated solar cells and hence efforts are made to tune the growth conditions of the films by incorporating Sn-rich additives (such as SnF_2_) in the precursor solutions to minimize the background doping levels in the perovskites.^56,57^ Nevertheless, Sn vacancies still account for one of the primary sources for the inherent doping and Sn^2+^ oxidation in Sn-containing perovskites and as such they affect the carrier recombination and transport properties of perovskites.^58^ Therefore, we then investigated the impact of such Sn vacancies on the migration of iodide ions in FAPb_0.5_Sn_0.5_I_3_ and FASnI_3_.

**Fig. 4:**
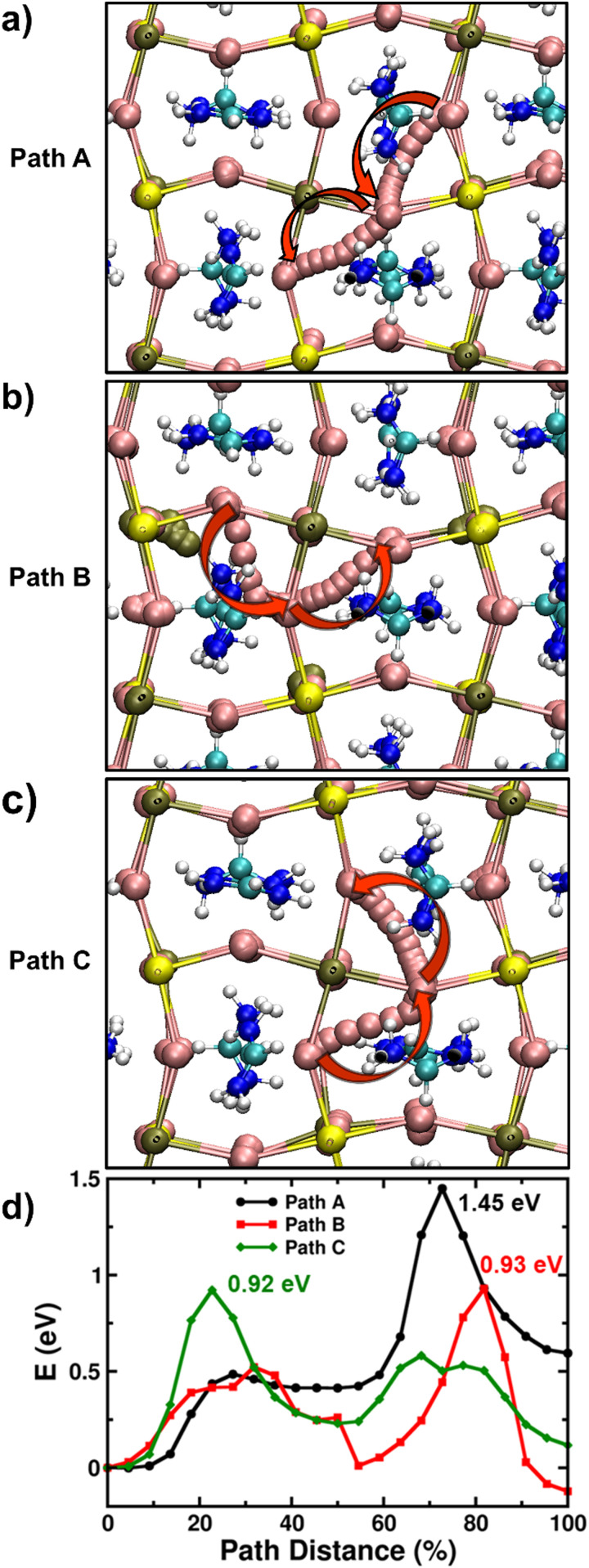
Ion migration pathways through atomistic *ab initio* simulations. (a)–(c) Inequivalent iodide migration paths (A, B and C) in FAPb_0.5_Sn_0.5_I_3_ involving the SnI_2_ Schottky-type defect comprised of a Sn vacancy and two I vacancies. Here, yellow, olive, pink, turquoise, blue and white spheres represent lead, tin, iodine, carbon, nitrogen and hydrogen atoms respectively. The red arrows point to the direction of iodide migration in the lattice. (d) Energy profiles for iodide ion migration *via* paths A, B and C in FAPb_0.5_Sn_0.5_I_3_.

It is found that the most stable SnI_2_ Schottky-type defect comprises of the Sn vacancy and two I vacancies (V_Sn_, 2V_I_) at adjacent sites rather than at well separated positions (Fig. S14, ESI†). Using the established computational framework for obtaining defect properties in halide perovskite materials,^59–61^ we calculate the SnI_2_ defect formation energies of 0.57 eV and 0.89 eV for FASnI_3_ and FAPb_0.5_Sn_0.5_I_3_ respectively, resulting in corresponding defect densities of 1.09 × 10^12^ and 1.34 × 10^7^ cm^−3^. Hence, it is highly likely that iodide ions diffusing over long distances (as in typical optoelectronic devices such as solar cells and LEDs) would inevitably encounter a SnI_2_ vacancy defect. Accordingly, with this Schottky defect cluster (V_Sn_, 2V_I_), we examined three distinct iodide ion migration pathways labelled A, B, and C (more details in Supplementary Note 3 in ESI†) in Fig. 4a–c, and their energy profiles are shown in Fig. 4d. For FAPb_0.5_Sn_0.5_I_3_, these A, B and C pathways involve different sequences of equatorial-axial type hops leading to energy barriers greater than 0.9 eV and to a rate-limiting ion migration energy for long-range diffusion of 1.45 eV. Following similar migration paths in FASnI_3_, we again find energy barriers for iodide ion migration greater than 0.9 eV and to a rate-limiting migration energy for long-range diffusion of 1.12 eV (Fig. S15, ESI†).

Table 2 summarises the rate-limiting migration barriers for iodide ions in the different model perovskite systems with and without the presence of Sn vacancies. Thus, our simulations clearly suggest that iodide migration near Sn vacancy defects in FAPb_0.5_Sn_0.5_I_3_ and FASnI_3_ face high energy barriers (>1.1 eV) compared to <0.5 eV for Pb perovskites due to severe local structural distortion, thereby reducing the mobility of halide ions. It is, however, important to note that due to the nature of *ab initio* simulations adopted in this study (*i.e.*, single crystal-like behaviour of model perovskite systems without the influence of effects such as grain boundaries or dynamic effects related to the ambient operational conditions of real devices), these differences in activation energy of iodide ions need to be understood in a qualitative manner for valuable comparison with the trends found from the experimental results. Moreover, first-principles calculations by Meggiolaro *et al.* suggests similar values of formation energy (and hence the density) of iodide vacancies irrespective of the choice of B-site metal.^62^ Therefore, the reduced migration of iodide ions in the presence of Sn may also correspondingly result in lower ionic conductivity.

We have also modelled the migration of Sn^2+^ cations through the available Sn vacancies in FASnI_3_ (Fig. S16, ESI†) and obtained a high migration barrier of 1.53 eV, which is even higher than the corresponding rate-limiting migration barrier of iodide ions. Therefore, similar to our previous work on MAPbI_3_,^8^ we find that the migration of B-site metal cations (Sn in this case) is associated with a large activation energy and hence unlikely to meaningfully contribute to the current–voltage hysteresis in solar cells.

Overall, we highlight the important role of Sn vacancies in arresting the migration of iodide ions in Sn-containing perovskites, in good accordance with our experimental observations of much slower ion diffusion in mixed Pb–Sn devices.

**Table 2:** Rate-limiting ion migration energies for long-range iodide ion transport in different model perovskite systems

Perovskite system	Ion migration energy (eV)
FAPbI_3_	0.45
FASnI_3_ (without Sn vacancies)	0.36
FASnI_3_ (in presence of Sn vacancies)	1.12
FAPb_0.5_Sn_0.5_I_3_ (without Sn vacancies)	0.47
FAPb_0.5_Sn_0.5_I_3_ (in presence of Sn vacancies)	1.45

## Conclusion

The impact of substituting Pb with Sn on the ion migration properties of halide perovskite optoelectronic devices has been investigated using a combination of experimental and computational techniques. Short circuit current loss obtained at lower scan rates (<50 mV s^−1^) indicates the prevalence of ion migration in both Pb and Pb–Sn perovskite solar cells, but the kinetics of ion transport are suppressed in mixed Pb–Sn systems as inferred from scan-rate dependent hysteresis measurements. These results are further corroborated by temperature-dependent impedance spectroscopy measurements performed on the fabricated devices at open circuit and under light illumination, which suggest a substantial lowering of ionic diffusion with the partial substitution of Pb with Sn. In addition, atomistic *ab initio* simulations highlight the role of Sn vacancies in increasing the iodide ion migration barriers (>1.1 eV) in Sn-containing perovskites due to severe local structural distortion, which corroborates and rationalises our experimental observations of much slower ion diffusion in mixed Pb–Sn perovskite solar cells. Overall, our findings can be generalized for a variety of Pb halide perovskite optoelectronic devices, where the benefit of Sn substitution in suppressing ionic migration effects may lead to enhanced operational stability and improved device architectures.

## Author contributions

K. D. conceived the idea and designed the experimental plan with supervision from S. D. S, M. S. I. and P. J. C.; K. D. optimized the processing of perovskite films and transport layers and accordingly fabricated all the devices for electrical characterization. K. D. performed and analyzed UV-Vis, XRD and PL measurements on the perovskite films. K. D. also carried out and analyzed standard and scan-rate dependent *J*–*V* measurements on perovskite solar cells. D. G. conducted DFT calculations on the migration barrier of iodides in different perovskite systems with assistance from P. D. and interpreted the results with M. S. I; M. P. and S. R. P measured impedance spectroscopy on perovskite solar cells and analyzed the data with P. J. C.; B. R. measured the top-view SEM of the perovskite films. S. P. S. provided useful inputs on the understanding of ionic migration in perovskite devices. K. D. interpreted all the data and wrote the first draft of the manuscript with detailed contributions from all the authors.

## Conflicts of interest

S. D. S. is a cofounder of Swift Solar. The remaining authors declare no competing interests.

## Supplementary Material

EE-017-D3EE03772J-s001
