# Supplementary material for: Substitution of lead with tin suppresses ionic transport in halide perovskite optoelectronics
Source: Energy Environ Sci. 2023 Nov 27;17(2):760–9. doi: 10.1039/d3ee03772j (PMC10805128; doi:10.1039/d3ee03772j)
Supplement: EE-017-D3EE03772J-s001 [file EE-017-D3EE03772J-s001.pdf]

Electronic Supplementary Material (ESI) for Energy & Environmental Science.
This journal is © The Royal Society of Chemistry 2023

Supporting Information

Substitution of Lead with Tin Suppresses Ionic Transport in Halide Perovskite Optoelectronics

Krishanu Dey,[1] Dibyajyoti Ghosh,[2] Matthew Pilot,[3] Samuel R. Pering,[4] Bart Roose,[5] Priyanka Deswal,[6] Satyaprasad P. Senanayak,[7] Petra J. Cameron,[3*] M. Saiful Islam,[8*] Samuel D. Stranks[1,5*]

[1]Cavendish Laboratory, University of Cambridge, Cambridge, UK

[2]Department of Materials Science and Engineering and Department of Chemistry, Indian Institute of Technology Delhi, Hauz Khas, India

[3]Department of Chemistry, University of Bath, Bath, UK

[4]Department of Materials, Loughborough University, Loughborough, UK

[5]Department of Chemical Engineering and Biotechnology, University of Cambridge, Cambridge, UK

[6] Department of Physics, Indian Institute of Technology Delhi, Hauz Khas, India

[7]Nanoelectronics and Device Physics Lab, National Institute of Science Education and Research, School of Physical Sciences, HBNI, Jatni, India

[8]Department of Materials, University of Oxford, Oxford, UK

Email for correspondence: sds65@cam.ac.uk, saiful.islam@materials.ox.ac.uk, chppjc@bath.ac.uk

Supplementary Note 1: Methods

I. Materials

All the starting perovskite precursors, viz. e.g. Formamidinium Iodide (FAI, GreatCell Solar Materials), Cesium Iodide (CsI, Sigma Aldrich, anhydrous, beads, −10 mesh, 99.999% trace metals basis), Lead Iodide (PbI_2, TCI UK Ltd.), Tin Iodide (SnI_2, Sigma Aldrich, anhydrous, beads, −10 mesh, 99.99% trace metals basis) and Tin Fluoride (SnF_2, Sigma Aldrich) were used without any further purification. Poly(3,4-ethylenedioxythiophene):poly(styrene sulfonate) (PEDOT:PSS) aqueous solution (Clevois P VP AI 4083) was purchased from Heraeus Co. Ltd. [2-(9H-Carbazol-9-yl)ethyl]phosphonic Acid (2PACz) was purchased from Tokyo Chemical Industry (TCI) Ltd. C_{60} and bathocuproine (BCP) were purchased from Creaphys GmbH and Ossila respectively.

II. Fabrication of perovskite films

All the perovskite film preparation was conducted in N_2-filled glovebox (H_2O < 0.1 ppm, O_2 < 0.1 ppm).

For $FA_{0.85}Cs_{0.15}PbI_3$ perovskite films, all the relevant precursors (FAI, CsI and PbI_2) were dissolved in appropriate ratios in a 4:1 (vol/vol) mixed solvent containing N,N-Dimethylformamide (DMF, Sigma Aldrich) and Dimethyl Sulfoxide (DMSO, Sigma Aldrich) and then left for stirring at room temperature for 2-3 hours. Following this, perovskite solutions were spin-coated on UV-ozone treated bare glass (for material characterization) or hole transport layer (HTL)-coated glass (for device fabrication) substrates using a two-step program: 1000 rpm (acceleration: 2000 rpm/s) for 10s and 5000 rpm (acceleration: 2000 rpm/s) for 30s, with chlorobenzene (antisolvent) dropped on the spinning substrates 5-10s before the end. The as-coated films were then annealed at 150 ^oC for 30 minutes.

For $FA_{0.85}Cs_{0.15}Pb_{0.5}Sn_{0.5}I_3$ perovskite films, all the relevant precursors (FAI, CsI, PbI_2, SnI_2 and SnF_2) were dissolved in appropriate ratios (a mixed solvent of DMF and DMSO (3:1 vol/vol) and then left for stirring at room temperature for 2-3 hours. Following this, perovskite solutions were filtered through 0.22 µm PTFE syringe filters and then spin-coated on UV-ozone treated bare glass (for material characterization)

or hole transport layer (HTL)-coated glass (for device fabrication) substrates at 5000 rpm (acceleration: 5000 rpm/s) for 50s, with anisole (antisolvent) dropped on the spinning substrates 25s before the end. The as-coated films were then annealed at 100 ^{o}C for 10 minutes. It is noted that a constant amount of SnF_2 (10 mol% with respect to the amount of Sn in the solution) was added in the solution to suppress the formation of Sn vacancies and accordingly the background doping in the fabricated Pb-Sn films.

III. Solar cell fabrication

Pre-patterned Glass/ITO substrates (10-15 Ω/sq, Kintec) were cleaned with 15 min ultrasonic bath in detergent (decon 90), water, acetone and isopropanol, followed by drying with a N_2 gun. After UV-ozone treatment of 15 minutes, PEDOT:PSS hole transport layer (HTL) was fabricated from an aqueous dispersion which was filtered through a 0.45 µm PVDF filter and then spin coated at 4000 rpm for 30s. The films were then annealed at 120 ^{o}C for 20 minutes and then taken immediately into the glovebox for subsequent deposition of perovskite films. For 2PACz HTL, 1 mg/ml solution in anhydrous ethanol (Sigma Aldrich) was spin coated on UV-ozone treated substrates in the glovebox at 3000 rpm for 30s, followed by annealing at 100 ^{o}C for 10 minutes. Perovskite films were then deposited on HTL-coated substrates using the above procedure for Pb and Pb-Sn based compositions. Subsequently, 20 nm C_{60}, 7 nm BCP and 120 nm Cu were sequentially deposited by thermal evaporation in a glovebox to complete the p-i-n devices.

IV. Material characterization

UV-vis-NIR spectrophotometry: These measurements were collected on a Shimadzu UV-3600Plus spectrophotometer using the transmission mode (without an integrating sphere).

Photoluminescence (PL): These measurements were performed using a linear PL setup with a 405 nm laser excitation. PL signals were collected in the reflection geometry with a Si detector (for Pb perovskites) and InGaAs detector (for Pb-Sn perovskites). A 450 nm long-pass filter was also used in the detection path to block the laser signal from reaching the detector.

X-ray diffraction (XRD): XRD measurements were performed in Bragg-Brentano geometry using a D8 Advance X-ray diffractometer (Bruker AXS, Karlsruhe, Germany)

with Cu-Kα source (1.5418 Å). Data for the experiments were collected in a locked-coupled 1D-mode for 2θ between 5° and 35°, with a step size of 0.01032°.

Scanning electron microscopy (SEM): The surface of the perovskite films was imaged using a Zeiss LEO 1550 FE-SEM apparatus, with a 2 kV acceleration voltage. It was ensured that the voltage levels for SEM measurement did not significantly impact the integrity of the perovskite thin films. Grain size analysis was performed using ImageJ software.

Solar cell measurements: A solar simulator from ABET Technologies (Model 11016 Sun 2000) with a xenon arc lamp was used to illuminate the solar cells (active area ~0.095 cm^2) at an intensity of 100 mW cm^{-2} ('1 sun' for AM1.5G spectrum) which were recorded using a Keithley 2635 source meter. Unless otherwise stated, all the *J*–*V* measurements were recorded in both reverse (open circuit to short circuit) and forward (short circuit to open circuit) directions in dark and under light at a scan speed of 100 mV s^{-1} with a voltage resolution of 50 mV. Silicon reference cells with different windows (such as KG5 and KG2) were used to calibrate the intensity of the solar simulator.

The distribution of the device performance (i.e. device statistics) for each condition is represented in the form of box plots, where the upper and lower bound of the box represents the upper quartile and lower quartile of the data respectively. The horizontal line splitting the box into two parts represents the median. On the other hand, the whiskers outside the box connect the upper quartile to the maximum of the data and the lower quartile to the minimum of the data.

External quantum efficiency (EQE) measurements: These measurements were performed using a Bentham PVE300 system equipped with xenon-quartz and tungsten halogen lamps and a single monochromator. During the measurements, we used a transformer mode (Bentham S400 474) with a frequency of ~ 300 Hz and a signal detection unit (Bentham S400 417) to measure the spectral response and EQE of our devices. A silicon reference cell was used to calibrate for the spectral mismatch.

Impedance spectroscopy measurements: These measurements were taken on a Solartron Modulab under 73 mW cm^{-2} illumination at open circuit between 1MHz and 1mHz with a 10 mV perturbation. A 470 nm blue LED was used as the illumination source. The temperature of the cell was controlled by a Peltier element which was

mounted into a gas tight chamber with a quartz window. The solar cell was mounted and electrically connected into the holder inside a glove box, and the chamber was filled with dry nitrogen. The chamber was then sealed, removed from the glove box and mounted in front of the LED.

V. Ab-initio calculations

The computational modelling of ion migration in halide perovskites is well developed and has been extensively applied by some of us and others.[1–3] The optimized structures and ground-state energies of pristine and mixed B-cation iodide perovskites are calculated using density functional theory (DFT) methods as implemented in the *ab initio* code CP2K.[4,5] All the simulations use Grimme-type dispersion corrections,[6] mixed Gaussian and plane-wave methodology, PBEsol-GGA as exchange-correlation functional,[7] and analytical dual-space pseudopotentials.[8] To model the mixed B-cation lattice, we use a 2×4×4 supercell (768 atoms; 64 formula units) of the tetragonal unit cell. Such a large supercell is necessary to model the structural defects and ion migration around those in the halide perovskite structure. For simulating $FAPb_{0.5}Sn_{0.5}I_3$, we have computed several ordered configurations of the B-metals. We find the structures where Pb and Sn are distributed in an ordered manner are more stable compared to structures where Pb or Sn form local clusters at adjacent B-sites. This is in agreement with previous experimental and computational results.[9,10] This ordering helps us to keep the number of inequivalent defect sites and subsequent ion-migration paths tractable within our computational framework. The activation energies for defect-induced ion migration processes are calculated by evaluating the energy difference between the diffusing species in their ground-state configuration and at the saddle point during the migration. We employ the climbing-image nudged elastic band (CI-NEB) method to determine the minimum energy path (MEP) for diffusion. The convergence criterion of the total energy is 1×10^{-6} eV. The force convergence is set up as 0.015 eV/Å in the CI-NEB calculations. The linear interpolation between the endpoints is used to prepare initial guessed MEPs using 18 intermediate images. The iodide defects or SnI_2 defects are separated by > 24 Å in these simulated models. Such large defect-defect distance guarantees negligible interactions between two defect-sites during modelling.

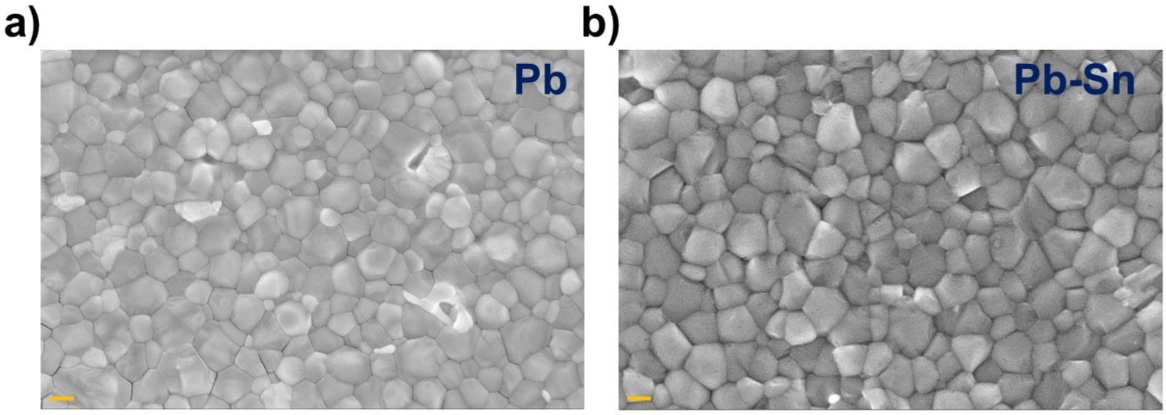

Figure S1. SEM images of a) Pb perovskite and b) Pb-Sn perovskite thin films on glass. The scale bar on the two images refers to 200 nm.

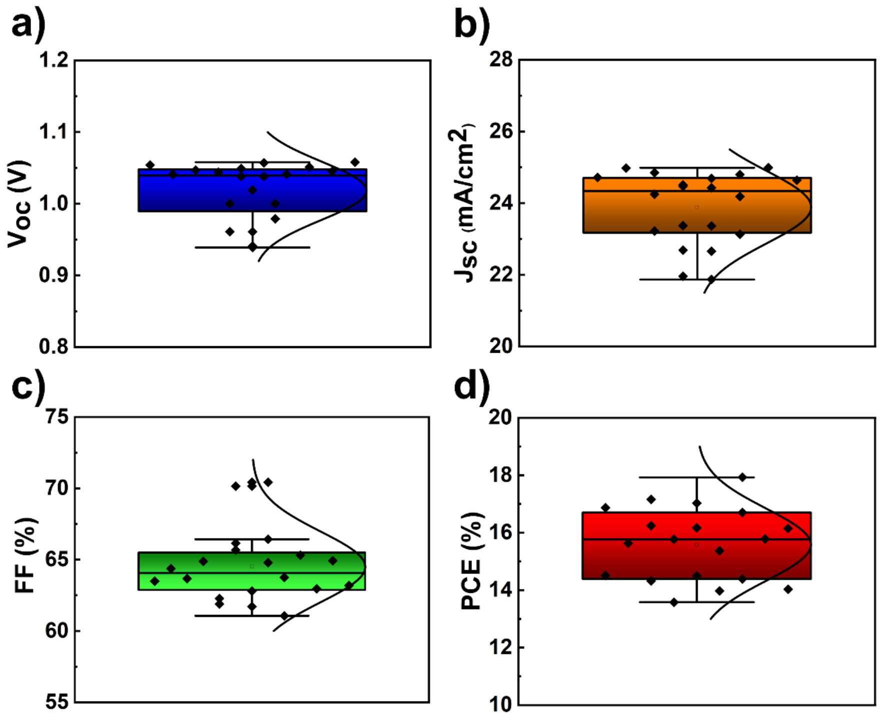

Figure S2. Statistics of photovoltaic parameters (V_{OC}, J_{SC}, FF and PCE) of Pb perovskite solar cells (2PACz as the HTL) extracted from 10 devices made across 3 batches. The data contains values from both reverse and forward scans.

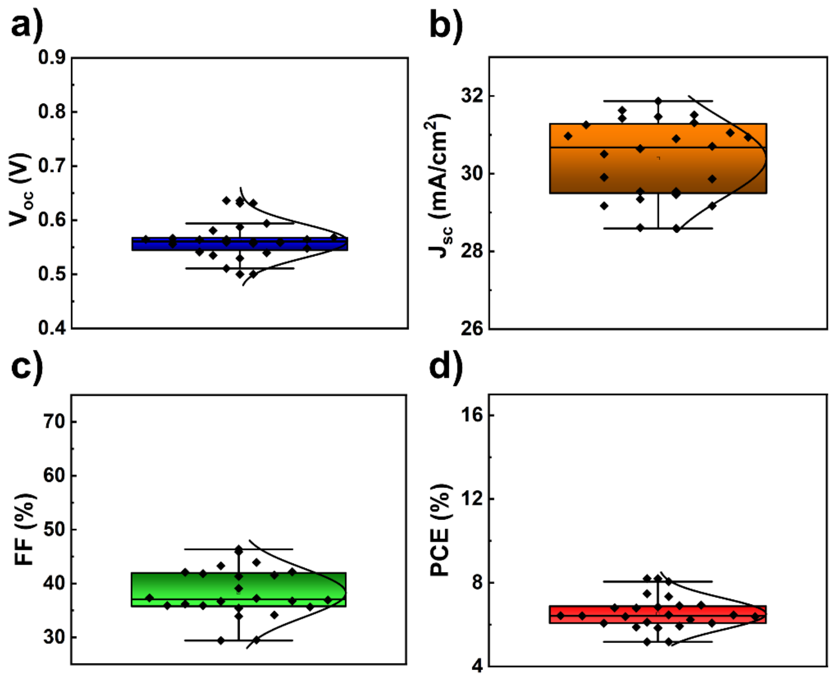

Figure S3. Statistics of photovoltaic parameters (V_{OC}, J_{SC}, FF and PCE) of Pb-Sn perovskite solar cells (2PACz as the HTL) extracted from 12 devices made across 2 batches. The data contains values from both reverse and forward scans.

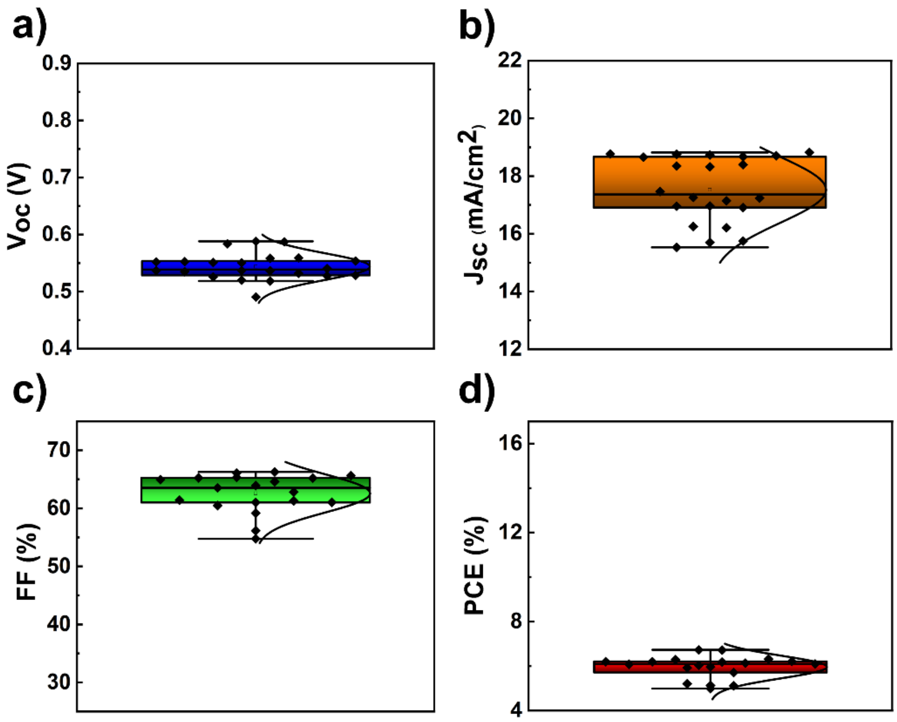

Figure S4. Statistics of photovoltaic parameters (V_{oc}, J_{sc}, FF and PCE) of Pb perovskite solar cells (PEDOT:PSS as the HTL) extracted from 10-12 devices made across 2 batches. The data contains values from both reverse and forward scans.

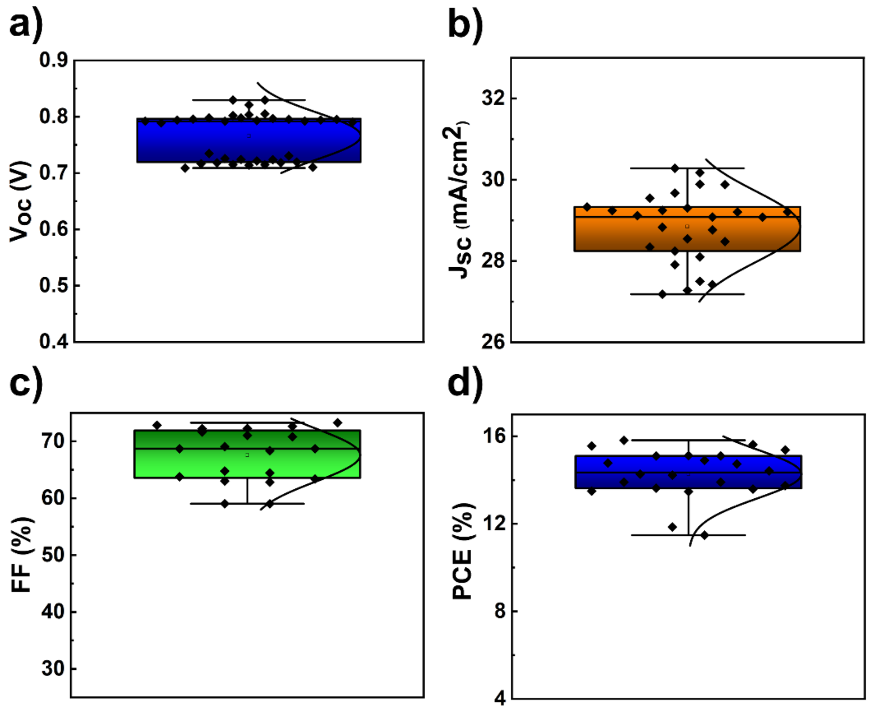

Figure S5. Statistics of photovoltaic parameters (V_{oc}, J_{sc}, FF and PCE) of Pb-Sn perovskite solar cells (405 nm absorber) with PEDOT: PSS as the HTL. The data is extracted from 10-15 devices made across 3 batches and contains values from both reverse and forward scans.

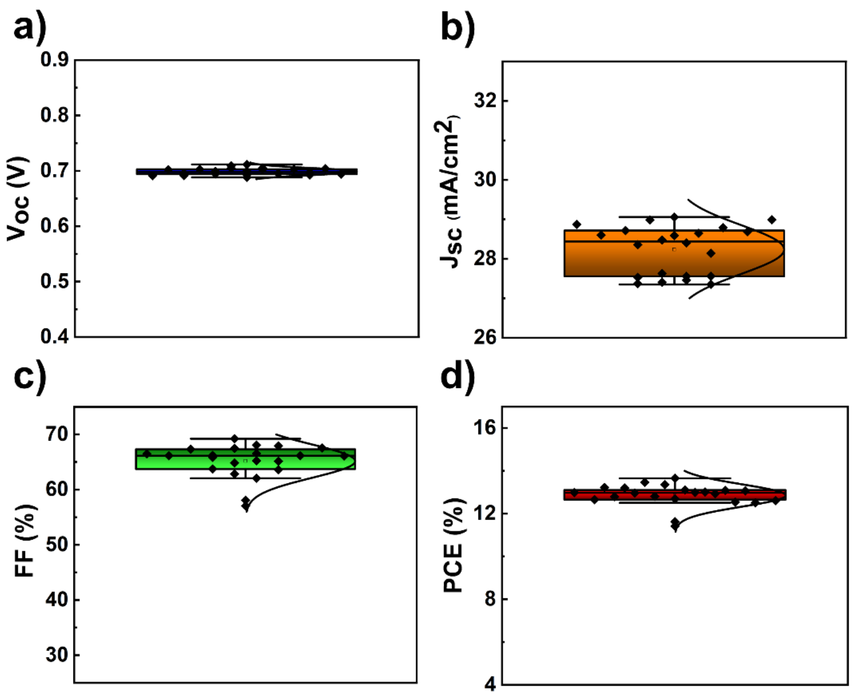

Figure S6. Statistics of photovoltaic parameters (V_{oc}, J_{sc}, FF and PCE) of Pb-Sn perovskite solar cells (made from 1.8M solution) with PEDOT: PSS as the HTL. The data is extracted from 11 devices made across 2 batches and contains values from both reverse and forward scans.

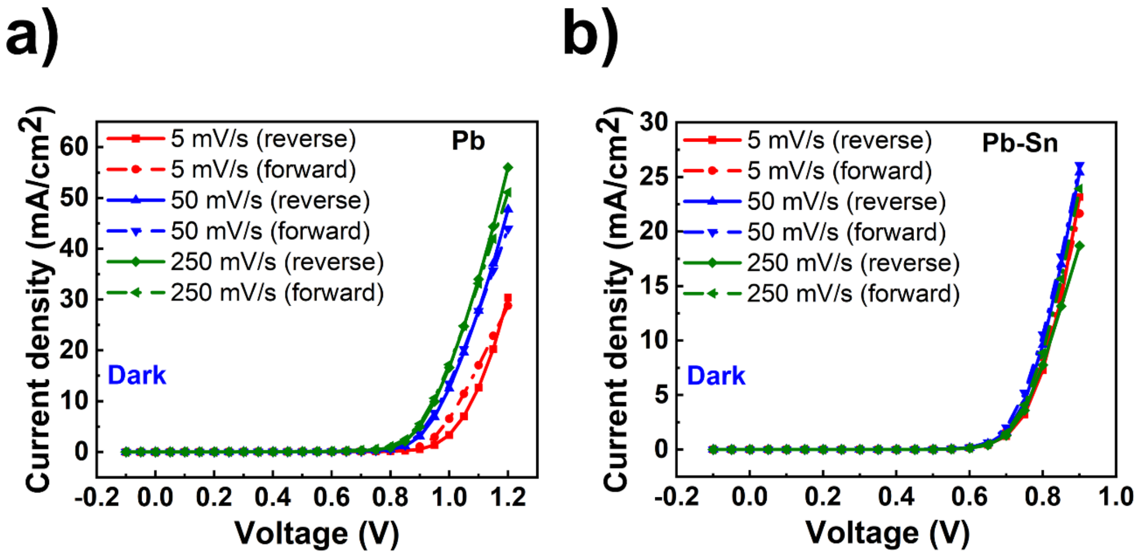

Figure S7. Dark J-V scans of (a) Pb and (b) Pb-Sn perovskite solar cells at three different scan rates: 5 mV/s, 50 mV/s and 250 mV/s.

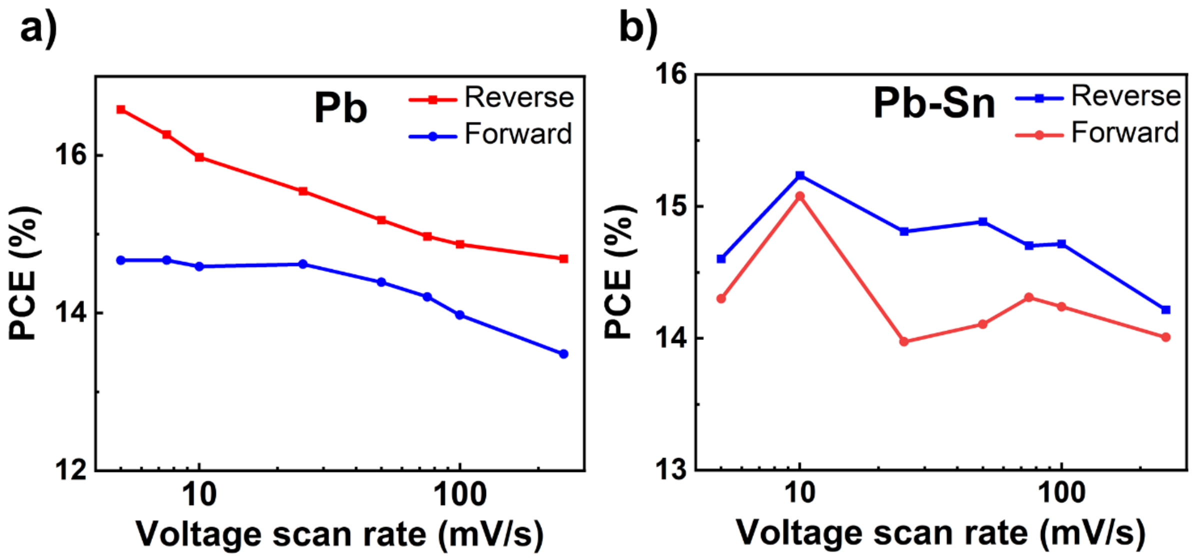

Figure S8. PCE of (a) Pb and (b) Pb-Sn perovskite solar cells as a function of scan rates for both reverse and forward scans.

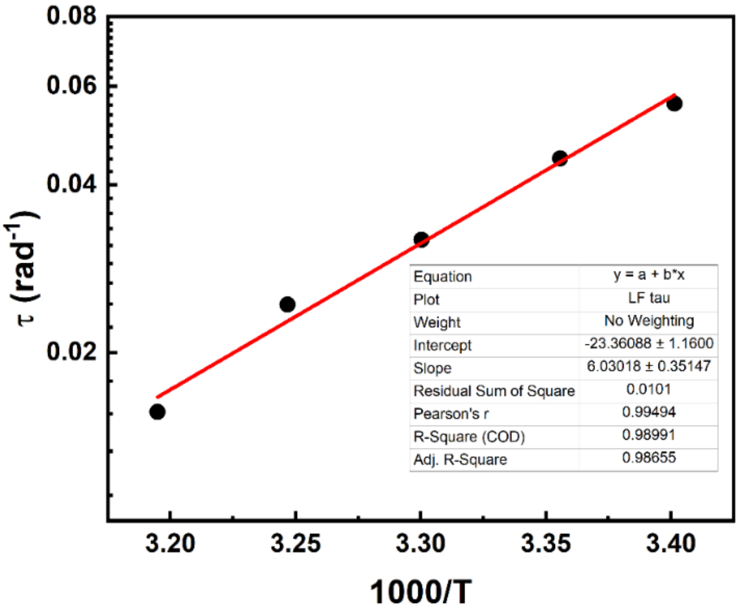

Figure S9. Arrhenius plot for the low frequency feature observed in the impedance response of Pb perovskite solar cell.

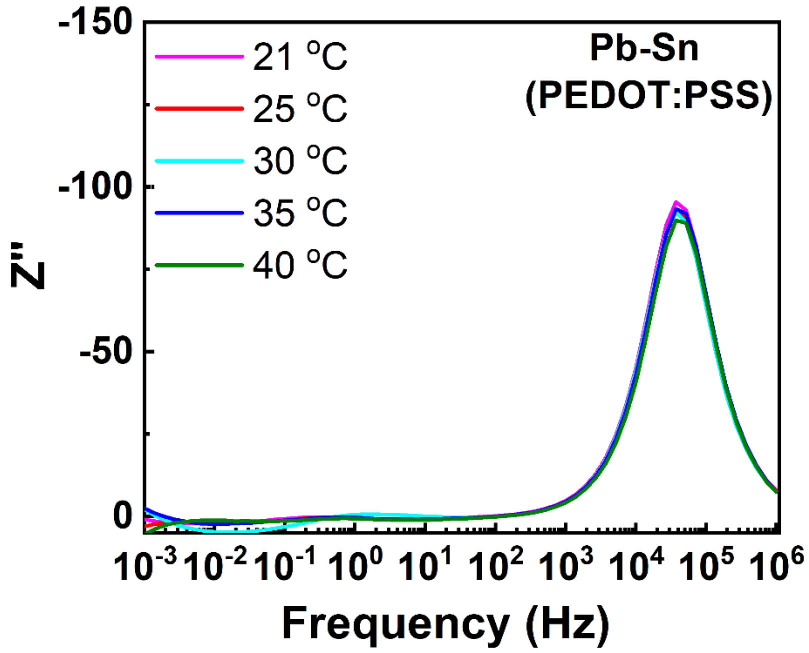

Figure S10. Plots of the imaginary impedance against frequency for Pb-Sn perovskite solar cell (with PEDOT:PSS as the HTL) within a range of temperatures (21 – 40 ^{o}C).

Supplementary Note 2: Kramers-Kronig analysis

The Kramers-Kronig test is a method of checking the validity of impedance results through the mathematical relation between the real and imaginary parts. Linear Kramers-Kronig analysis calculates the difference from the experimental data when the real component is calculated from the imaginary component, and *vice versa*.[11] The graphs in **Figure S11** show these differences as a residual %. A residual of less than 1% signifies a valid impedance result. **Figures S11**a-b show that there is low deviation (<1%) in the real and imaginary parts of the spectrum for both Pb and Pb-Sn devices down to 0.1 Hz, thereby rendering the data valid within these frequencies.

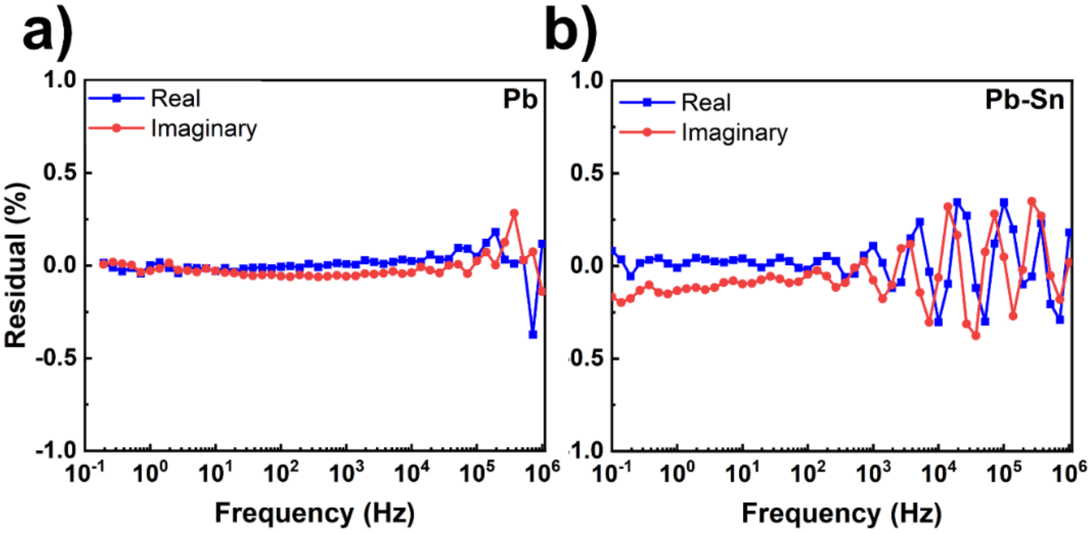

Figure S11. Kramers-Kronig test for validating the impedance measurements of a) Pb and b) Pb-Sn perovskite solar cells.

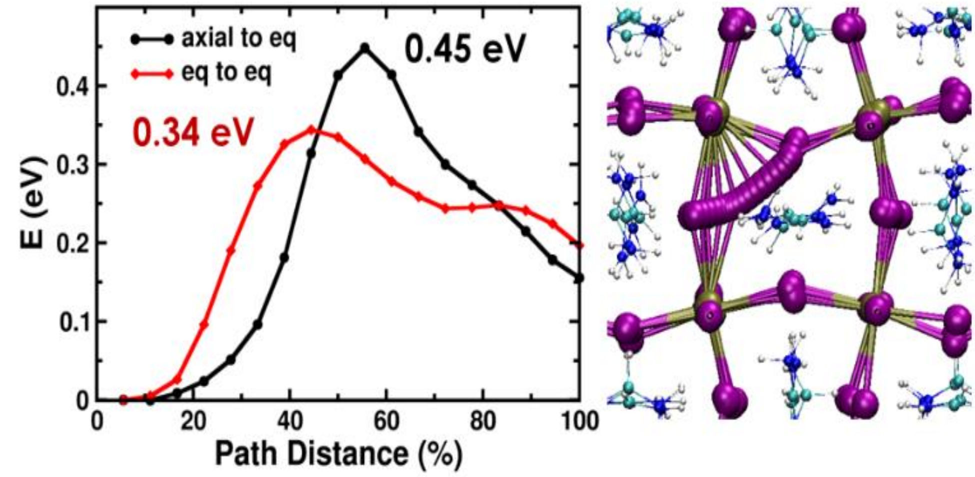

Figure S12. The energy barriers for vacancy-assisted iodide migration in $FAPbI_3$ through axial-to-equatorial and equatorial-to-equatorial pathways.

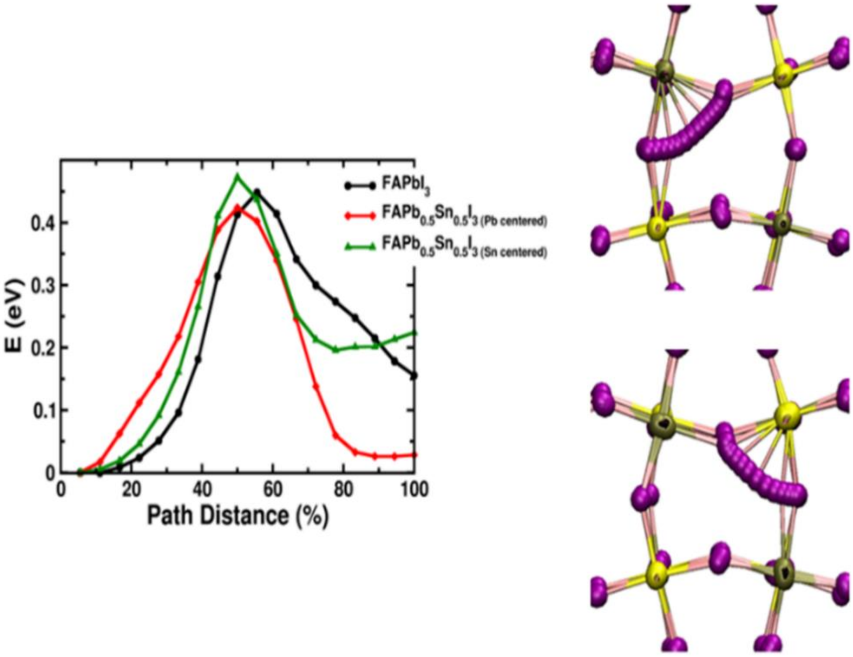

Figure S13. The energy barriers for iodide migration in $FAPb_{0.5}Sn_{0.5}I_3$. The inequivalent migration paths are presented on the right.

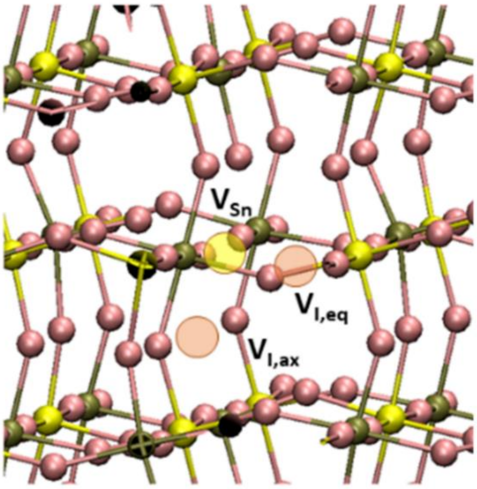

Figure S14. The most stable configuration of the Schottky-type SnI_2 defect of one Sn vacancy and two I vacancies in $FAPb_{0.5}Sn_{0.5}I_3$.

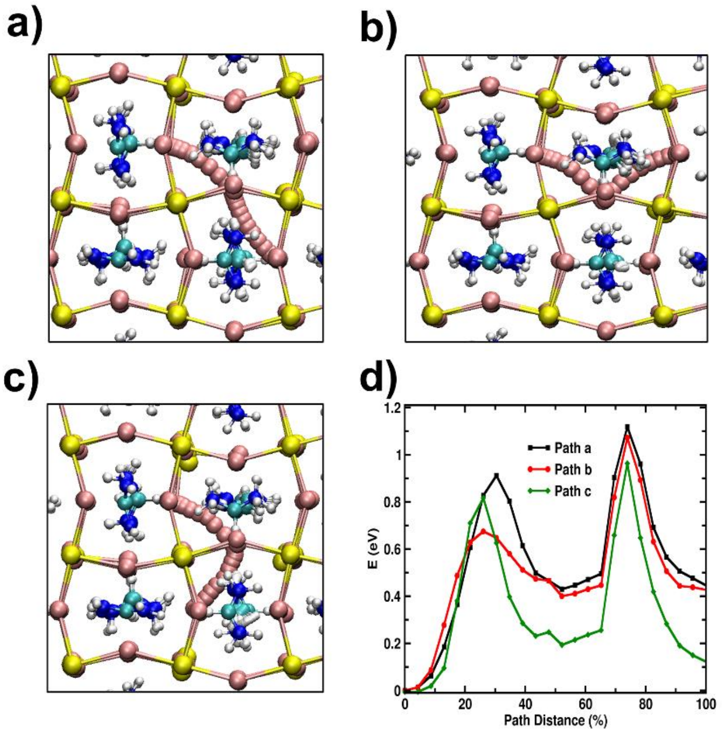

Figure S15. The simulated iodide migration paths (a-c) involving the SnI_2 Schottky type defect site in $FASnI_3$. Along these inequivalent paths, iodide ion migrates with different activation energy. (a) An axial iodide migrates to equatorial iodide vacancy first and then hops to another axial iodide vacancy (Path a), (b) iodide ion first hops from the axial to equatorial vacancy site and then migrates from that equatorial site to the vacant axial site (Path b), (c) iodide ion migrates from the axial site to the vacant equatorial site and then hops to another vacant axial site (Path c), (d) The energy barriers for different inequivalent pathways for iodide ion to diffuse through SnI_2 defect clusters in $FASnI_3$.

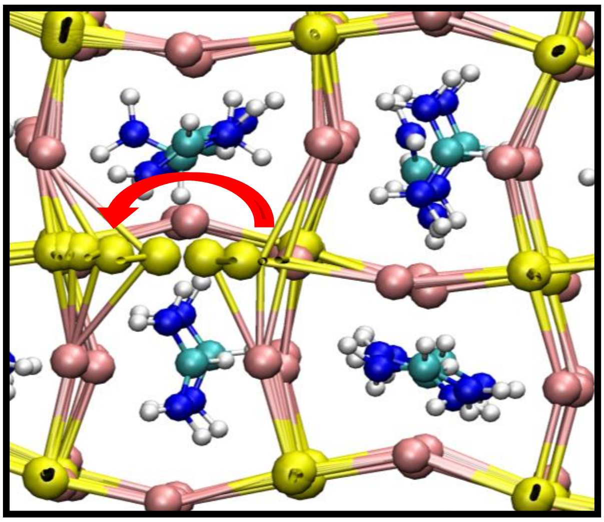

Figure S16. The simulated migration pathway for mobile Sn^{2+} ions through the Sn vacancies in $FASnI_3$.

Supplementary Note 3: Pathways for iodide diffusion in $FAPb_{0.5}Sn_{0.5}I_3$

Path A: Here, an axial iodide migrates to equatorial iodide vacancy first and then hops to axial iodide vacancy. In this process, the iodide ion initially breaks an Sn-I axial bond to migrate. During the second hop, the ion breaks a Pb-I bond and forms an Sn-I axial bond. The migration barriers are 0.48 eV and 1.44 eV, respectively for these two sequential hopping.

Path B: Here, the migrating iodide ion first hops from the equatorial to the axial vacancy site and then migrates from that axial site to the vacant equatorial site. Migration barriers for these two hopping are calculated as 0.52 eV and 0.92 eV respectively.

Path C: Here, the diffusing iodide ion migrates from the axial site to the vacant equatorial site and then hops to another vacant axial site. Simulating the migration path, we find that the initial iodide hop has a large activation energy barrier of 0.92 eV.

References

1. D. W. Ferdani, S. R. Pering, D. Ghosh, P. Kubiak, A. B. Walker, S. E. Lewis, A. L. Johnson, P. J. Baker, M. S. Islam and P. J. Cameron, *Energy Environ. Sci.,* 2019, **12**, 2264–2272.

2. C. Eames, J. M. Frost, P. R. F. Barnes, B. C. O'Regan, A. Walsh and M. S. I., *Nat. Commun.,* 2015, **6**, 2–9.

3. J. Haruyama, K. Sodeyama, L. Han and Y. Tateyama, *J. Am. Chem. Soc.,* 2015, **137**, 10048–10051.

4. J. VandeVondele, M. Krack, F. Mohamed, M. Parrinello, T. Chassaing and J. Hutter, *Computer Physics Communications,* 2005, **167**, 103–128.

5. T. D. Kühne, M. Iannuzzi, M. D. Ben, V. V. Rybkin, P. Seewald, F. Stein, T. Laino, R. Z. Khaliullin, O. Schutt, F. Schiffman, D. Golze, J. Wilhelm, S. Chulkov, M. H. B.-Hashemian, V. Weber, U. Bornstnik, M. Taillefumier, A. S. Jakobovits, A. Lazzaro, H. Pabst, T. Muller, R. Schade, M. Guidon, S. Andermatt, N. Holmberg, G. K. Schenter, A. Hehn, A. Bussy, F. Belleflamme, G. Tabacchi, A. Glob, M. Lass, I. Bethune, C. J. Mundy, C. Plessl, M. Watkins, J. VandeVondele, M. Krack and J. Hutter, *J. Chem. Phys.,* 2020, **152**, 194103.

6. S. Grimme, J. Antony, S. Ehrlich and H. Krieg, *J. Chem. Phys.,* 2010, **132**, 154104.

7. J. P. Perdew, A. Ruzsinszky, G. I. Csonka, O. A. Vydrov, G. E. Scuseria, L. A. Constantin, X. Zhou and K. Burke, *Phys. Rev. Lett.,* 2008, **100**, 136406.

8. S. Goedecker, M. Teter and J. Hutter, *Phys. Rev. B.,* 1996, **54**, 1703–1710.

9. A. Goyal, S. McKechnie, D. Pashov, W. Tumas, M. V. Schilfgaarde and V. Stevanovic, *Chem. Mater.,* 2018, **30**, 3920–3928.

10. G. E. Eperon, T. Leijtens, K. A. Bush, R. Prasanna, T. Green, J. T.-W. Wang, D. P. Mcmeekin, G. Volonakis, R. L. Milot, R. May, A. Palmstrom, D. J. Slotcavage, R. A. Belisle, J. B. Patel, E. S. Parrott, R. J. Sutton, W. Ma, F. Moghadam, B. Conings, A. Babayigit, H.-G. Boyen, S. Bent, F. Giustino, L. M. Herz, M. B. Johnston, M. D. McGehee and H. J. Snaith, *Science*, 2016, **354**, 861–865.

11. M. Schonleber, D. Klotz and E. Ivers-Tiffee, *Electrochimica Acta,* 2014, **131**, 20–27.
